# Determinants of Pregnancy Outcomes After Assisted Reproductive Therapy: A Sample From the West Bank, Palestine

**DOI:** 10.7759/cureus.43011

**Published:** 2023-08-06

**Authors:** Hasan Arafat, Diaeddin Qamhia, Husam Maqboul, Abdulsalam Alkaiyat

**Affiliations:** 1 Cancer Care Center, Augusta Victoria Hospital, Jerusalem, PSE; 2 Medical Laboratory Department, Specialty Arab Hospital, Nablus, PSE; 3 Emergency Department, Al-Watani Hosptial, Nablus, PSE; 4 Biomedical Sciences Department, An-Najah National University, Nablus, PSE

**Keywords:** gynecology, obstetrics, follicle stimulating hormone, assisted reproductive technology, infertility

## Abstract

Background

Infertility is a public health issue with a significant impact on the well-being of affected couples.

Aim

This paper aims to detect the determinants of pregnancy and their outcome after assisted reproductive therapy (ART) in a sample of Palestinian society.

Methods

A retrospective observational study was carried out at Razan Medical Center for Infertility. Subjects were assigned into twelve categories based on the type of infertility (primary versus secondary), the cause of infertility, and the treatment modality. Age at marriage, age at presentation, duration of infertility, in addition to regularity of menstruation, were also studied. Biochemical pregnancy was considered the endpoint for the purpose of the analysis.

Results

We reviewed the files of 459 subjects diagnosed with infertility. 79.74% had primary infertility while 20.26% had secondary. 28.85% were found to be infertile due to anovulation, 2.86% due to endometriosis, 16.74% attributed to male factor, and 3.3% had tubal damage. 13.43% of cases were multifactorial while 34.80% were idiopathic. Four biochemical markers were assessed in our study: thyroid-stimulating hormone (TSH) (*x̄*=2.32±2.46), luteinizing hormone (*x̄*=6.71±4.90), follicle-stimulating hormone (FSH) (*x̄*=6.59±6.11), and human prolactin (*x̄*=41.88±6.50). The menstrual cycle was regular in 70.58% of subjects, in contrast to 29.41% with irregular cycles. The female mean age at presentation was 22.76±5.58, while their mean age of marriage was 22.76±4. The mean duration of infertility was 3.97±6.87 years. Patients were treated via three modalities - ovarian stimulation (2.2%), intrauterine insemination (29.58%), and intracytoplasmic sperm injection (68.21%) - with a success rate of 51.85%. Of the studied factors, only diagnosis, follicle-stimulating hormone, and treatment modality had a significant impact on the outcome, with *p-*values of 0.040, 0.003, and <.0.001, respectively.

Conclusions

There is a strong relationship between diagnosis prior to intervention, level of FSH, and treatment modality and successful outcome of ART.

## Introduction

Infertility is defined as failure to achieve clinical pregnancy after 12 months or more of regular, unprotected sexual intercourse according to the World Health Organization (WHO) [1]. For many couples, the lack of ability to have children profoundly impacts their social, physical, and sexual well-being [2]. Infertility is a public health issue for couples of reproductive age, affecting about 72.4 million women aged 20-44 with an estimated prevalence of 9% [3]. Infertility can be classified into primary and secondary; while primary infertility refers to couples who have never achieved pregnancy, secondary infertility refers to those who have conceived at least once before, but now are unable to [4].

As a result of the huge impact of infertility on individual and social well-being, many epidemiological studies reviewed and analyzed the factors that have been attributed to infertility to provide guidance for the prevention of infertility. Conventionally, these parameters include the age of marriage, body mass index (BMI), duration of infertility, obstetric history, psychological stress, menstrual regularity, environmental factors, and occupational exposures [5].

All steps must occur correctly during ovulation, fertilization, and implantation to achieve pregnancy. Several factors can cause a disturbance at any step, whether due to female or male reasons. Female reasons leading to infertility included: hormonal disorders, polycystic ovarian syndrome, premature ovarian failure, endometriosis, fallopian tube obstruction, congenital uterine anomalies [6], and complications of medical diseases (diabetes and hypothyroidism) [7,8]. Male infertility is usually due to sperm abnormalities or hormonal imbalance [2,9].

Palestinian women's fertility rate is considered one of the highest in the world, with an average of 4.38 births per woman [10]. Unfortunately, no studies were conducted to detect the determinants of pregnancy outcomes in Palestinian women. This study aims to detect the characteristics and pregnancy outcomes after assisted reproductive therapy (ART) at Razan Medical Center for Infertility.

## Materials and methods

Study design and settings

This is a retrospective cohort study. Data were collected from the electronic records of the patients who presented to the Razan Center for the management and evaluation of infertility between 2015-2018. The study included patients from Razan branches in three major cities in the West Bank of the Palestinian Territories - Nablus, Ramallah, and Bethlehem - which serve the whole Palestinian population in the West Bank.

Inclusion and exclusion criteria

All cases that met the WHO definition of infertility, defined as the inability to conceive despite regular, unprotected sexual intercourse for 12 consecutive months, were included. Those who have never conceived before were defined as primary infertile, while those who had at least conceived once prior to the start of the study were categorized as secondary. Cases that presented for follow-up of normal pregnancy, gender selection, frozen embryo transfer as well as records with missing data were excluded. In addition, cases that did not meet the WHO definition of infertility in terms of duration and inability to have regular sexual intercourse were also excluded too.

Sample and data collection

A total of 459 files were reviewed according to the above-mentioned inclusion and exclusion criteria. Four categorical variables were studied. The first variable was the type of infertility - whether primary or secondary. The second variable was the underlying cause of infertility - male factors, anovulation, endometriosis, or anatomical tubal damage. Patients who had more than one cause were labeled multifactorial, while those in whom no specific diagnosis was reached after extensive workup were labeled idiopathic.

Patients who were found to have polycystic ovarian syndrome (PCOS), ovarian insufficiency (whether premature or age-related), and hypogonadotropic hypogonadism were all grouped under the category of anovulation. Endometriosis was assigned a separate category due to the ambiguity of the exact mechanism by which endometriosis causes infertility.

The third categorical variable studied was the regularity of the menstrual cycle - the response was either 'regular' or 'irregular' based on the patient's answer and subjective assessment. The fourth categorical variable was the treatment modality. The studied modalities were - ovarian stimulation (via clomiphene citrate, follitropin alfa, or dydrogesterone), intrauterine insemination (IUI), and intracytoplasmic sperm injection (ICSI).

Continuous variables studied were - the biochemical markers of infertility, including follicle-stimulating hormone (FSH), luteinizing hormone (LH), thyroid-stimulating hormone (TSH), and human prolactin (hPRL). The sociodemographic factors studied were - age at presentation, age at marriage, and the duration of infertility.

Data analysis

The primary endpoint of the study was biochemical pregnancy, defined as a positive serum β-human chorionic gonadotropin (β-hCG) test. The predictors investigated for association with a positive biochemical pregnancy were diagnosis, level of TSH, day two LH, day two FSH, hPRL, age at marriage, age at presentation, duration of infertility, regularity of the menstrual cycle, and modality of treatment. The endpoint and predictors were described as means and standard deviations (SD) for quantitative variables and as absolute values and percentages for categorical variables. Differences in predictor variables according to the presence or absence of biochemical pregnancy were described using Chi-squared test and t-test, as appropriate. Statistical significance was defined as a two-sided p-value <0.05. All data were analyzed using the STATA Data Analysis and Statistical Software, version 14 (StataCorp, College Station USA).

## Results

The total number of infertile females who met the inclusion criteria was 459. The mean age of the sample was 26.81 (SD ± 5.84 years, range 17 to 45 years). The duration of infertility was less than 5 years in 390 females (80.2%), and was 5 years or more in 96 females (19.75%). Three hundred and sixty-six (79.7%) females had primary infertility while the rest (20.2%) had secondary infertility. Details of the demographic data of the females are listed in Table 1.

**Table 1:** Details of the demographic data of the females SD: standard deviation.

Parameter	Result
Mean Age ± SD in Years	26.81 ± 5.84
Mean Duration of Infertility ± SD in Years	3.97 ± 6.87
Primary Infertility, N (%)	366 (79.7)
Secondary Infertility, N (%)	93(20.2)

One hundred thirty-one cases (28.85%) were diagnosed as infertile due to anovulation, disregarding whether anovulation was due to PCOS, hypogonadotropic hypogonadism, or premature ovarian failure. Seventy-six cases (16.74%) were attributed to male factors, 15 cases (3.3%) were due to tubal damage, 13 cases (2.86%) were due to endometriosis, 158 cases (34.8%) were idiopathic, defined as patients with no apparent cause of infertility despite having a regular menstrual cycle, and 61 cases (13.43%) being multifactorial (infertility attributed to more than one of the cases mentioned above). These findings are summarized in Table 2.

**Table 2:** Type of infertility, diagnosis, menstrual status, and modality of treatment for the total sample, pregnancy, and non-pregnancy outcomes with p-values (N=459) IUI: intrauterine insemination, ICSI: intracytoplasmic sperm injection.

		Total N (%)	Pregnant N (%)	Non-Pregnant N (%)	p-value
Type of Infertility	Primary	366 (79.74)	189 (51.64)	177 (48.36)	0.268
	Secondary	93 (20.26)	54 (58.06)	39 (41.94)	
Diagnosis	Anovulation	131 (28.85)	65 (49.62)	66 (50.38)	0.040
	Endometriosis	13 (2.86)	10 (76.92)	3 (22.08)	
	Male Factor	76 (16.74)	46 (60.53)	30 (39.47)	
	Tubal Damage	15 (3.30)	11 (73.33)	4 (26.67)	
	Idiopathic	158 (34.80)	73 (46.20)	85 (53.80)	
	Multifactorial	61 (13.43)	36 (59.02)	25 (40.98)	
Menstrual Status	Regular	324 (70.58)	168 (51.85)	156 (48.15)	0.469
	Irregular	135 (29.41)	75 (55.56)	60 (44.44)	
Treatment Modality	Ovarian Stimulation	10 (2.20)	4 (40.00)	6 (60.00)	<0.001
	IUI	134 (29.58)	33 (24.63)	101 (75.37)	
	ICSI	309 (68.21)	201 (65.05)	108 (34.95)	

Three hundred and sixty-six (79.74%) cases were of primary infertility, defined as the inability to conceive despite 12 months of regular, unprotected vaginal intercourse; 324 (70.58%) had regular menses; 10 cases (2.2%) were treated with ovarian stimulation with hormonal treatment constituted; 134 (29.58%) of cases were treated with IUI, while the most of the cases (68.21%) were treated with ICSI. Table 2 shows the categorical variables and outcomes with p-values. Table 3 lists the mean and SD for biochemical markers of infertility, age at marriage and presentation, and duration of infertility for the total sample, pregnancy, and no pregnancy outcomes.

**Table 3:** Mean and SD for biochemical markers of infertility, age at marriage and presentation, and duration of infertility for the total sample, pregnancy, and no pregnancy outcomes SD: standard deviation, TSH: thyroid-stimulating hormone, LH: luteinizing hormone, FSH: follicle-stimulating hormone, hPRL: human prolactin.

	Total Sample Mean (±SD)	Pregnant Mean (±SD)	Non-Pregnant Mean (±SD)	p-value
TSH	2.32 ± 2.46	2.25 ± 2.38	2.38 ± 2.54	0.592
LH	6.71 ± 4.90	6.71 ± 4.91	6.71 ± 4.89	0.995
FSH	6.59 ± 6.11	6.06 ± 5.72	7.12 ± 6.50	0.003
hPRL	41.88 ± 6.50	36.94 ± 5.78	46.82 ± 7.21	0.282
Age at Marriage	22.76 ±5.58	22.49 ± 4.82	23.03 ± 6.34	0.308
Age at Presentation	26.81 ± 5.84	26.75 ± 5.30	26.88 ± 5.30	0.805
Duration of Infertility	3.97 ± 6.87	3.97 ± 0.29	3.98 ± 0.60	0.99

## Discussion

In our study, we evaluated the effect of hormones, demographic determinants, as well as treatment modality on biochemical pregnancy among a female sample from Palestine. Despite Palestine being a developing country, its fertility pattern is considered distinct compared to that of other, surrounding developing nations in that the high fertility rate coexists with a high level of education and a low level of infant mortality. Moreover, due to the special political situation in Palestine, fertility is not considered a mere biological reality, but a political one - Palestinian women desire to have more children as a means of resistance against the continuous displacement and human loss due to ongoing conflict [10].

In our study, we found a statistically significant relationship (p-value of 0.003) between FSH level pre-in vitro fertilization (IVF) and biochemical pregnancy, which was in agreement with several studies in which FSH level was found to be a significant negative predictive factor of clinical pregnancy [11,12]. LH levels were not statistically significant (p-value of 0.995) with regard to the biochemical pregnancy test**.** This finding was opposed to some studies that showed that the mean serum concentration of LH was significantly low in couples who conceive in comparison to couples who did not [13,14]. The other studied hormones, namely, TSH and hPRL (p = 0.592, 0.282, respectively), did not have a statistically significant relationship with the outcomes and the success of the pregnancy trial, which was in agreement with previous studies that showed no significant difference between the two groups in the serum concentration of TSH and hPRL [13]. In addition, we found a very strong relationship between the modality of treatment and biochemical pregnancy.

As we predicted, the pre-treatment diagnosis had a statistically significant relationship (p = 0.04) with the outcome, but in clinical practice, it is rare for child-bearing-age women to have an isolated reproductive disorder, as most of them suffer from multiple conditions [15]. This means that multiple, coexisting reproductive disorders should be taken into account when studying pregnancy outcomes. Our finding coincides with another study that summarizes all reproductive disorders that could affect the pregnancy outcome in women of childbearing age [16]. Our results revealed that whether the patient had not had a previous pregnancy (primary infertility) or failed to conceive after one or more successful pregnancies (secondary infertility) had no statistical significance on the outcome (p = 0.268). Whether the patient had a regular menstrual cycle or not did not impact the outcome of treatment (p = 0.469). Chronological parameters of the patient did not impact the outcome of treatment as well - the mean age at marriage was relatively the same when comparing that of women with successful outcomes compared to those with a failure result (23.03 years of age vs. 22.49 years), the relationship, as expected, was not statistically significant (p = 0.308).

The age of presentation did not significantly affect the outcome (p = 0.805), as expected by the close means of the age of successful vs unsuccessful cases (26.75 vs. 26.88). The duration of infertility is measured as the number of years between getting married and presenting to the infertility center. Those who presented before fulfilling one year of infertility were told to come back after fulfilling the required duration. As there was no difference between successful and unsuccessful cases regarding the duration of infertility (both groups had a mean of 3.98), the relationship was statistically insignificant (p = 0.99). These findings might be attributed to the fact that most of the women studied belonged to the same age category, and further studies, including more women of a wider spectrum of age categories, should be conducted. Regarding the modality of treatment, a very strong relationship was found between the type of treatment and the success of the cycle, as the rate of success for intracytoplasmic sperm injection (ICSI) was significantly higher compared to intrauterine insemination and ovarian stimulation (68.21% vs 29.58% vs 2.20%), with a p-value of 0.001.

This clinical audit is considered the first at Razan Medical Center for Infertility, as it will help modify and enhance clinical practice and patient care. In addition, the large sample size helped define the actual relationships between different determinants. Being a retrospective cohort study, its results are considered relatively strong and reliable. Our study was limited by the high number of incomplete files, manifested by the fact that of the total of 459 infertile females enrolled in the study, only 291 completed their investigations, giving a rate of 63.5% of adherence to follow-up. The insufficient data were in the biochemical markers of infertility, including FSH and LH.

## Conclusions

We found a strong relationship between FSH levels with the success of treatment. The underlying diagnosis significantly affected the success rate. Our most important finding was the extremely significant relationship between the ICSI technique and positive outcome, as it was superior to both IUI and ovarian stimulation in achieving a positive end result. We recommend starting with ICSI for eligible patients to save their time and effort in achieving their desired results. However, this remained an obstacle given how expensive ICSI is compared to IUI. Furthermore, we recommend testing FSH hormone as routine for patients who present to the IVF center.
